# “Mtoto Wa Nyoka Ni Nyoka,” The Child of a Snake is a Snake: A Narrative Analysis of Adverse Childhood Experiences and Perpetration of Interpersonal Violence Among Men in Dar es Salaam, Tanzania

**DOI:** 10.1177/0886260521997443

**Published:** 2021-03-05

**Authors:** Susannah Zietz, Lusajo Kajula, Sandra Martin, Beth Moracco, Meghan Shanahan, Suzanne Maman

**Affiliations:** 1 Duke University, Durham, NC, USA; 2 UNICEF Office of Research-Innocenti, Florence, Italy; 3 University of North Carolina at Chapel Hill, Chapel Hill, NC, USA

**Keywords:** Adverse childhood experiences, intimate partner violence, peer violence, narrative analysis, life history

## Abstract

Childhood exposure to adversity, including abuse and neglect, is consistently found to be a predictor of intimate partner violence (IPV) and peer violence (PV) perpetration in adulthood. The purpose of this study is to qualitatively examine factors that may facilitate or impede the use of violence among those who have been exposed to adversity early in life. We are particularly interested in protective experiences or environments for these participants. The qualitative data were analyzed through thematic coding and narrative analysis of participant life histories.

We found three salient themes: (a) parental acceptance and early attachment is protective for coping with stress with intimate partners in adulthood; (b) certain key life turning points can provide a protective context against violent behavior in adulthood; and (c) poverty in adulthood compromises one’s ability to cope with stress and anger in adulthood.

Our findings contextualize the different factors that may affect the behavior of perpetration of interpersonal violence among high-risk men in Dar es Salaam who have been exposed to adversity in childhood. These findings provide important information on the risk and protective factors for interpersonal violence spanning from childhood to adulthood. This study highlights the importance of child development interventions in this situation, both for the primary prevention of child adversity and for promoting resilience and mitigating the effects of childhood adversity that put men at risk for perpetration of interpersonal violence in adulthood.

## Introduction

Both exposure to adverse childhood experiences (ACEs) and interpersonal violence such as intimate partner violence (IPV) and peer violence (PV) are public health and human rights problems with far reaching inter-generational consequences for the well-being of children, families, and communities. Childhood exposure to adverse experiences such as abuse and neglect, violence, or non-violent family dysfunction (e.g., loss of a parent or caregiver, household member in jail, alcoholic household member, etc.) is consistently cited as a strong predictor of violence perpetration in adolescence and adulthood (Fulu et al., 2013; Gil-Gonzalez et al., 2008; Jewkes, 2002; Roberts et al., 2010; Wilkins et al., 2014). Exposure to ACEs may not be limited to individual incidents, but a pattern of polyvictimization throughout the life course (Finkelhor et al., 2007). Once children become poly-victims, they maintain an elevated risk of victimization over their lives (Finkelhor et al., 2007). In order to inform primary prevention of all types of violence and to coordinate and integrate responses, there has been a call for research seeking to understand shared risk and protective factors of violence, so as to influence the prevention of multiple connected forms of violence such as perpetration of IPV, PV, and child abuse and neglect (Wilkins et al., 2014).

There is a growing body of research on how protective factors, characteristics that reduce the negative impact of a risk factor on problem outcomes, particularly around parenting, can attenuate the relationship between ACEs and interpersonal violence perpetration. A variety of domains of parenting (e.g., maternal warmth, positive parenting, maternal social support) have been found to differently relate to the adjustment of children exposed to adversity, depending on the environmental, social, and cultural contexts (Capaldi et al., 2012; Fong et al., 2017; Gorman-Smith et al., 2004).

Urban Tanzanian men are a critical population to target with violence prevention interventions. According to a UNICEF survey, approximately 30% of women and 13% of men ages 13–24 reported experiences of sexual violence prior to 18, and three-quarters of both men and women in this population experienced physical violence before the age of 18 (UNICEF & CDC, 2011). Findings from research studies in Dar es Salaam also indicate that a significant proportion of men perpetrate IPV and PV (Mulawa et al., 2018; Fleming & Jacobsen, 2010). A sample of men in four districts in Dar es Salaam found that 27.6% of men reported perpetration of some form of IPV in the past 12 months (Mulawa et al., 2018). Physical fighting is a common occurrence among adolescent boys age 13–15 in Dar es Salaam. One study found that 45% of male students reported participating in a physical fight one or more times during the past 12 months (Fleming & Jacobsen, 2010).

With a population growth rate of about 8% per year, Dar es Salaam is one of the fastest growing cities in sub-Saharan Africa. Similar to other urban areas of sub-Saharan Africa, there is a concentration of economic, health, and social issues in Dar es Salaam. Rapid growth in the private sector is increasing economic inequality and fragility in the city. The city now has areas of high and moderate wealth interspersed with areas of high poverty (Kessides, 2007; Owens, 2014). According to a United Nations estimate, 70% of the city’s population lives in informal settlements, many without running water or basic services. Many of these residents are migrants from rural areas, known locally as “upcountry.” It is estimated that half of the city’s annual population growth of 5.8% (from 2002 to 2012) may be attributed to internal migration (NBS, 2013, 2016). Disruption in social ties due to migration to Dar es Salaam has been linked to a shift in the locus of child rearing from the community to the nuclear family (Leshabari & Kaaya, 2005).

Corporal punishment by caregivers is normative in Tanzania and is often seen as an essential feature for effectively raising a child. Frankenberg et al. (2010) found that corporal punishment is common in informal settlements in Dar es Salaam. Caregivers described different discipline strategies, which depend on factors such as the capacities of the caregiver, the age and gender of the child, and the nature of the perceived behavioral transgression of the child. The strategies include “beating with care,” including controlled and moderate discipline that is accompanied by warmth from the caregiver, “as if beating a snake,” which is conceptualized as overly harsh physical punishment, and the “non-care of non-beating,” used to describe caregivers who neglect their children through not disciplining them (Frankenberg et al., 2010).

The pathways from adversity in childhood to subsequent perpetration of interpersonal violence are complex and multifaceted. Current analyses of these pathways often lack contextual information on the interaction of different risk and protective factors over time. The goal of this paper is to qualitatively examine, among high-risk men in Dar es Salaam, how different adverse experiences in childhood affect use of interpersonal violence as an adult. We are particularly interested in protective experiences or environments for these participants.

## Methods

### Study Setting

Participants for this study were purposively sampled from the control group of a cluster randomized control trial that examined the efficacy of a multilevel intervention to reduce sexually transmitted infections and IPV among networks of men in Dar es Salaam, Tanzania (Kajula et al., 2015). For the larger trial, the study team identified venues called “camps,” which are spaces where social network members spend time every day, often for several hours. These camps provided unique access to social networks of high-risk young men who would otherwise be difficult to reach, since most are not in school or formally employed. Men in camps provide social support to one another, including contributing money when there was an illness or death in the family, engaging in small business enterprises together, and talking to one another about personal problems (Yamanis et al., 2010). Men in the parent trial reported high rates of childhood adversity. A total of 68% reported physical abuse, 71% report emotional abuse, and 50% report sexual abuse before the age of 18 (Mulawa et al., 2018).

The parent study took place in four populated and impoverished wards (equivalent to US census tract) within one of the five districts in Dar es Salaam. (Kajula et al., 2015; NBS, 2016).

### Theoretical Framework

Life course theory provides a framework for studying the social, cultural, and developmental pathways and trajectories among current study participants (Elder et al., 2003). Each of the five principles of life course theory informed the research questions, instruments, and analysis of this study.

1. **Life-span development.** It is important to understand contextual changes over time and their impact on individual lives. The study was guided by a life history perspective, where men narrated their experiences from their childhood to their current circumstances.
2. **Agency.** In the study, an emphasis was placed on the agency (capacity to make decisions and set goals) by which individuals construct their own “subjectively meaningful and coherent biographies in response to objectively contingent life courses” (Heinz, 2016, p. 22).
3. **Time and place.** Participants are shaped by the historical context of urbanization and internal migration in Tanzania, shifting the locus of child rearing from the community to the nuclear family.
4. **Timing.** We analyzed the narrative life histories from this study through the lens of the timing of events in a participant’s life.
5. **Linked lives.** The interpretation of a participant’s experience of adversity could be shaped by the lives of others, particularly through the social norms of corporeal punishment in Tanzania at the time.

### Study Sample

Participants purposively selected for this qualitative study were at least 18 years of age, had at least one sexual partner within the past 12 months and had experienced specific patterns of adversity in childhood that put them at the highest risk of perpetrating interpersonal violence in adulthood. These high-risk classes were identified using a previously conducted “person centered” quantitative analysis of patterns of ACEs and the relationship between these patterns and the subsequent perpetration of interpersonal violence in adulthood (Zietz et al., 2020). Participants selected for this qualitative study were at the highest risk of perpetration of IPV and/or PV based on the quantitative analysis that preceded this qualitative study (Kajula et al., 2015). Additionally, within this high-risk group, we selected participants who had either reported perpetrating IPV and/or PV or did not reported perpetrating IPV and/or PV in the quantitative study. Of all the participants in the study, 42% (10) reported perpetrating IPV in the past 12 months and 33% (8) reported perpetrating PV in the past 12 months. This enabled us to focus on potential risk and protective factors for interpersonal violence perpetration among men who experienced similar patterns of ACEs in childhood.

Twenty-four participants were interviewed from September to November 2017, at which point we determined that we had reached saturation in key topics and themes.

### Data Collection

The in-depth interview guide was developed based on a priori areas of interest based on the theoretical and empirical literature on ACEs and interpersonal violence perpetration. Particularly, we were interested in how men perceived acceptance or rejection in the relationship with their caregivers and how they coped with stress and anger in adulthood. Within the life-span development/life history perspective, their perception of acceptance or rejection in the relationship was seen as a key way to contextualize the violence that they experienced through the lens of their current situation and agency. For instance, guiding interview questions included: (a) “Can you tell me about the household where you grew up?” (b) “As a child, what did you think about the way you were disciplined? What do you think about how you were disciplined as a child now?” (c) “Give me an example of a stressful time during your childhood?” (d) “What are some of the things that you find stressful in your life now? What has it been like to feel stressed? What do you think specifically caused this stress?”

Interviews were conducted by two male Tanzanian researchers with extensive experience in qualitative research methods and working in the study community. The researchers also received training on ACEs and interpersonal violence research. The in-depth interviews were conducted in Kiswahili. Each interview ranged from 20 to 50 minutes. All participants in the in-depth interviews received 10,000 TZS (~$4.5) as reimbursement for transportation costs to the study field offices.

The study was conducted in accordance with the guidelines for safe and ethical study for research on gender-based violence that was developed for the United Nations Multi-Country Study on Men and Violence (Fulu et al., 2013). It was explained in both the recruitment script and consent process that the study sought to follow-up with participants on topics from the previous study survey they completed to learn more about their experiences, particularly stress, conflict, and/or violence in childhood and adult relationships. As part of the consent process, research assistants informed participants that the topics covered are highly personal and potentially distressing and that they are free to terminate the interview at any point or skip any questions that they do not want to answer and that the data collected will be held in strict confidence. Procedures were developed for active referral to free psychological counseling at Muhimbili National Hospital for participants who appeared distressed by the interview content. The study was approved by the Institutional Review board at The University of North Carolina at Chapel Hill and Muhimbili University of Health and Allied Sciences. Research clearance was obtained from the Tanzania Commission for Science and Technology.

In-depth interviews were conducted, audiotaped, transcribed in Kiswahili, and translated into English. Research assistants also recorded field notes immediately after completing each in-depth interview to document the content and process of the data collection. The field notes supplemented the transcripts as data sources.

### Narrative Analysis

We used an analytic approach that was guided by Maxwell and Miller’s (2008) work of integrating, categorizing, and connecting strategies. Categorizing analysis is based on comparisons among participants. The most common categorizing strategy is thematic coding and sorting by code. In contrast, connecting analysis focuses on trajectories within individual narratives (Maxwell & Miller, 2008).

In the first step, the first author immersed herself in reading and rereading transcripts and summaries and writing in-depth memos in order to identify emerging themes. She then compared these emerging themes to a priori themes developed when conducting the literature review and creating the interview guide. She compiled the themes into a separate codebook for the in-depth interviews, coded the data using inductive and deductive codes, and conducted the categorizing analysis using Atlas.ti Version 8 (Friese, 2014). Then, she re-read transcripts and field notes and wrote 1–2-page narrative summaries of seven participants whose life stories seemed to reflect predominant themes. These summaries holistically described the context in which participants experienced adversity in childhood and their perpetration of interpersonal violence in adulthood.

To identify key aspects of the participant’s stories related to the trajectory from childhood adversity to coping with stress and anger in adulthood, the first author analyzed the narrative summaries within the context of the five principles of life course theory. Key aspects of the trajectories across participants were compared and contrasted to understand how different contextual experiences over time can result in differences in coping and subsequent perpetration of interpersonal violence. She compared the experiences of the men in the narratives, breaking it up into early-childhood, mid-childhood, adolescence, young adulthood, and adulthood. She then summarized which trajectories were similar and different at each time and also across all of the periods. She then wrote memos in order to understand emerging themes about what were the key differences in men’s lives that seemed to affect their stress, coping, and aggression in adulthood.

Since the analysis for this study was based on transcripts translated from Kiswahili to English, the first author discussed emerging findings and questions with the research assistants and transcriptionist. She also shared findings with our study collaborator (co-investigator on the intervention study) in Tanzania to elicit feedback and refine interpretation.

## Results

In presenting findings in the Results section, pseudonyms are used for all participants. First, we present a summary of participant demographics, experience of ACEs, and perpetration of interpersonal violence. Second, we discuss three themes that explain the pathways between exposure to ACEs and interpersonal violence perpetration. Third, two narrative case studies are used to further illustrate the themes.

### Participant Characteristics

Interviews were conducted with 24 participants with varying IPV and PV perpetration histories. Demographics from the IDIs and endline survey are reported in Table 1. When asked about conflict with their current or last partner and peers in the qualitative interviews, there were differences from what the participants reported in the quantitative study. Only 9 of the men talked about violence towards their current or last partner in the interviews (compared to 13 men who reported it in the quantitative survey); 3 men talked about physical fights with their peers (compared to 11 men who reported it in the quantitative survey); and 12 said that they did not engage in either of these behaviors (compared to 11 who reported not engaging in these behaviors in the quantitative survey.

**Table 1.** Participant Demographics.

	*N* = 24
*From IDIs*	
Age	Mean 28 (18–39)
Loss of Parent before 18	13 (54%)
Lived with someone who was not his parent before 18	16 (67%)
Fathered at least one child	9 (38%)
*From endline survey*	
Perpetrated any IPV in past 12 months	10 (42%)
Perpetrated any PV in past 12 months	8 (33%)

The three major themes are (a) parental acceptance and attachment, (b) turning points, and (c) the role of poverty in shaping men’s ability to cope with stress and conflict. To preserve the holistic structure of the life history narratives, we then demonstrate our findings through recounting the story of two participants through the lens of these themes.

### Parental Acceptance Promotes Positive Coping with Stress and Conflict

Sixteen participants (67%) lived with a caregiver who was not their parent before the age of 18. Most of these were in informal kinship-based fostering. Reasons for this included (a) a parent dying, (b) parents divorcing, (c) illness in the family, (d) poverty, particularly parents not being able to provide for the participant’s basic needs, and (d) behavioral concerns including other relatives taking in a badly behaving child to discipline him. Most participants felt that they were not accepted as part of the new family that they were living in, whether it was with extended family members or a divorced parent and his new partner. One participant, Saleem, age 29 at the time of the interview, lived with extended family members because his parents were young and could not provide for him. He described this as “mapamba nje,” which can be translated as “clothes out,” indicating that one is constantly on the move, keeping his/her clothes outside to make it easier to move when needed.

“Mapamba nje” is a [phrase] which… I like to use that he is a child who didn’t benefit from his real proper parents’ upbringing… So “mapamba nje” means today you will find me living with my aunt and tomorrow I will be shifted to another place and study in another school.

When asked about his experience of affection in childhood, he said,

As a child, you get pain in your heart as you feel that parents don’t love you… I feel that I have not met them, and they have not come to see me and there is no communication between them to see how I, their child, [is] going on. So I felt that my parents didn’t love me… When you live with grandmother… she slapped you and you feel that with your father and mother, these things would not have happened…. I felt that my mother did not love me so I felt they bore me by accident, so that I can annoy my relatives and they can annoy me.

From comparing the patterns of summarized trajectories of participants, we found that participants like Saleem who reported living without parental affection were generally less able to cope with stress and anger in adulthood compared to participants who received affection from their parents. They also seemed to perceive the behavior of their peers and intimate partners with suspicion. For example, Hakim, who is profiled later in the paper, grew up with his uncle and felt less valued than his uncle’s children. He describes how he often copes poorly with his emotions when his wife does not respect his authority, such as not informing him before she goes somewhere. According to Hakim, a few weeks before the interview:

We quarreled because I found her absent from home when I came back from work. I found the door locked while I was tired and I wanted to eat and go and rest. Even if she went to see a friend in a nearby street still, she didn’t tell me where she was. So I got angry and when I entered inside, I decided to give her two to three slaps. So she got angry and went back to her parent’s home.

In contrast, despite extensive adversity in childhood, including temporarily living away from one’s parents, parental acceptance and attachment seemed to be protective for coping with stress and conflict with intimate partners and peers in adulthood. Particularly, related to the life course perspective of timing, attachment early in life seemed to be the most protective. Participants, who described feeling loved by their parents or guardians and feeling provided for, generally, were able to cope with stress and anger in adulthood without fighting. Thirteen of the fourteen participants who described feeling loved by their parents or guardians coped with stress and anger in adulthood without using violence or other risk-taking behaviors such as using drugs or alcohol. Additionally, only 3 of the 10 participants who reported feeling unloved or rejected by their parents coped with stress and anger in adulthood without using violence or other risk-taking behaviors.

### Key Life Turning Points Provide Context for Violent Behavior

Corresponding to the life course principle of life-span development, a number of participants faced contextual changes in their life circumstances, particularly their environment, which had the potential to be a protective or a risk factor for violent behavior later in life. Examples of this included moving out of an urban environment, joining the military, having a parent die, being in jail for a short period of time, and experiencing a serious health issue. For instance, Abdul found himself socializing with criminals and finding the criminal behavior to be “normal and a good thing.”

Abdul’s parents sent him to live with his uncle for 2 years in a different social environment. During this time, Abdul also developed a chronic issue with his leg that limited his mobility. Abdul considers the move and the issue with his leg to be what protected him from the danger of socializing with gangs in the area.

I usually tell one of my friends that if my parents would not have shifted me from those areas, and if I didn’t get these leg problems, I don’t know where I would have been today. I think I would have already died.

However, relocating was not always protective. Men often moved into situations where they were exposed to more ACEs. For instance, when Adamu’s stepfather passed away, his mother did not have money for him to continue with school to attend Form 3 (around 15-year old). His mother went to ask his biological father for money to support Adamu’s schooling, but his father fell ill and could not work so his mother ended up taking care of him. After the turning point of dropping out of school, Adamu left home and lived with friends who engaged in behaviors such as alcohol use, smoking marijuana, and stealing.

When father died, I stopped going to school for lack of school fees… and I decided to leave and I went and stayed far away from my parents for about 6-7 months with my friends in a ghetto… we were making some plans on what we should do, but life failed me and I went back home.

### The Role of Poverty in Shaping Men’s Ability to Cope with Stress and Conflict

One theme across all participants was the ways in which poverty compromised participants’ abilities to cope with stress and anger in adulthood. This can be contextualized with the life course principle of time and place, where in Dar es Salaam, there are limited economic opportunities and a shortage of affordable housing (Kessides, 2007). When asked about what stresses them in adulthood, almost all of the participants mentioned economic factors such as lacking money to provide for their families, being unemployed, not having sufficient income to move out of the family home or to start a business. However, the effect of these economic concerns on their use of violence seems to be compounded among participants who experienced a lot of adverse experiences while growing up.

For instance, Jamil felt a lot of stress because he was constantly thinking of how to financially support his wife and children. Growing up, Jamil lived with his mother, stepfather, and five half-siblings. He passed his secondary school exams but was not able to continue in school because his mother became ill and was very sick for almost 2 years. He experienced violence from his stepfather. He described being beaten with sticks by his stepfather when he misbehaved but felt worse when his stepfather was harsher towards his half-siblings. In adulthood, Jamil felt his “heart beats changing” when he was stressed about money and has a hard time sleeping. He started to use alcohol and described using it to escape the stress of his daily life.

I usually don’t like to have stress that’s why at other times I drink alcohol… If it is in the evening, I can decide to drink alcohol so that I can sleep.

He also described being quick to fight when he got overwhelmed with stress.

I am always very tolerant, but I usually get very angry easily and fail to tolerate and I show it by my reactions… If it is fighting, then no one can intervene in our fight because I fail to speak as I get a sudden stammering and my legs tremble very much. So I fail to talk and do the actions.

### Participant Narratives

In this section, the three main findings of the qualitative analysis are presented through the lens of two narratives of men from the in-depth interviews. We selected two participants as case examples because they included clear and detailed illustrations of the three themes presented above that were common to many of the 24 participants. Rashid’s story points to the role of parental attachment and acceptance and key life turning points while Hakim’s story demonstrates how economic stressors act as a catalyst for violence among men who have been exposed to adversity in childhood.

### Rashid: Strong Parental Attachment and Acceptance and Key Life Transition Points

Rashid is 34-year old and unemployed. He met his wife 14 years ago when he and his now wife were both supposed to be in secondary school but were unable to enroll due to lack of money. They married when she dropped out of school.

Rashid’s mother, a police officer, raised him along with his eight siblings. His mother was the sole source of parental affection and the family’s livelihood. Many participants mentioned that their parents showed affection through providing them with food and clothing. In contrast to this, Rashid stated that he knew that his mother loved him, despite her not being able to provide him with these things.

We loved each other and I never felt that money was an issue; because if I stayed hungry, I knew for sure that mother had nothing. And still we lived in peace and I had never felt that there was something different.

According to Rashid, his mother tried to instill good values in her children and led by example.

She united us with our neighbors and when she cooked food, she invited neighbors’ children to eat with us, she taught us so… She built us in a certain good ethics, which could assist us to live well with other people in future.

However, Rashid also experienced harsh discipline from his mother and uncle.

Our mother was very harsh… I used to observe her fighting with my brothers and sisters and I was afraid. But on my side, I didn’t get troubles with my mother very much. But there was our uncle who is called Mr. Hassan. He had a lot of time to spend at home with us because he rented a room in our neighborhood. He used to punish us very much before our mother came back from work… He could say because you have offended me, I will punish you… He can go to pick a stick, or he could hold you up by the cheeks and lifts you into the air and you hang in the air until he is satisfied and until you have accepted your mistake and it pains very much.

In general, Rashid was resigned to the discipline, especially when his mother gave it.

You cannot be beaten and feel happy, but you had to accept the results because it is the parent who has punished you and you have to listen to what she is telling you and follow what she wants.

Rashid’s mother passed away when he was in Standard 1 (around 7-year old). After this, his eldest brother married and moved away, taking the youngest children in the household with him. His sister, who was in Standard 7 at the time (around 14-year old), stayed at their childhood house in order to protect it from squatters. Though both Rashid and his sister were young, he stayed with her because no one in the extended family was able to care for him. During this time, he spent his time hanging out with friends, smoking marijuana, and stealing small things. Eventually, his grandfather visited them and decided to take Rashid and his sister to live with him in a rural village.

So when my grandfather came, he found that we were in [a] bad environment and I was the only younger child left there with my sister who was also not grown up, though she had finished standard 7. So grandfather said my sister cannot take care of me and [if I continue living there], I will fail to continue studying.

Rashid believed that this move to the village protected him from getting into trouble with his peers in the neighborhood.

Many of my friends with whom we were together that time when I was growing up from 9–10, many of them have died. So I think when my grandfather took me to the village… those things which I had started to join in like hooliganism, I left them and I started a new life in the line which my mother had laid a foundation for me.

Rashid was unable to consistently attend school in the village. He was often sent home because his grandfather could not pay school fees or because he was late to school because he was working. Rashid had to farm, collect firewood, cook, and take care of his grandfather. One day, he reached school late and was severely beaten by a teacher.

She took a big piece of wood thicker than my arm and she told me to bend over and touch the ground, so the backbone was protruded out. And she beat me with that big piece of wood until the backbone went back inside. So the school lessons did not continue because I had to be carried to the hospital. I got treatments and the pains were released but until today if someone knocks me on the back, I feel pains… When I think about it, I feel so bad because I see that I was abused.

However, there were other teachers at the school who understood his circumstances and supported him in getting an education.

My teachers who knew my life history and because of my ability in class they were telling me that “what will help you is education because from your life history your grandfather is now aged and your mother has died you don’t have any more assistance, education is the one which will be of assistance to you.” That’s why I was making efforts to study hard because my teachers advised me very much to put efforts in education, even neighbors and relatives who were aware about education they advised me to continue with education telling me that where you have reached [adulthood] without education you will suffer in your future life because your mother has died.

Rashid passed secondary school exams and had obtained sponsorship to attend Form One. At that point, his father, who was not a part of his life thus far, invited him to come and live with him. Thinking that he could live with his father and attend school in Dar es Salaam, he moved in with his father and stepmother. However, his stepmother convinced his father not to spend the money to educate him, claiming that Rashid was not his child.

While my peers were going to boarding schools and coming back during holidays, though my father had the financial ability, I was not going to school, I was just staying at home like a house boy, [and] my heart was very painful.

Rashid experienced another life transition when his father eventually fell ill and died. He decided to live in the house that his mother left him. At the time of the interview, he was 34-year-old, still living in his mother’s house, and was unemployed. He felt like he missed out on a lot of opportunities in life. Rashid sometimes deals with his stress through drinking alcohol. However, he mentioned that:

Most of the time I calm down and look at the direction of my life. I engage myself in small different activities in order to forget thoughts and push [on with] life. Because I am hard working, I struggle to make life go on.

Rashid has not had physical conflict with his peers and mostly has positive interactions in his social group.

Sometimes exchange of words happens. But we never reached a point of fighting or if someone wants to fight with another one the rest intervene, and we prevent the fight.

Despite his experience of being abused and engaging in high-risk behaviors with groups of boys, such as stealing and drinking alcohol, Rashid does not perpetrate IPV or PV and deals with his stress and anger in other ways. When asked to describe his childhood, Rashid immediately focused on the period in which he lived with his mother and the love she felt for him. Unlike other participants that has life histories similar to Rashid and went on to be violent in adulthood, Rashid mentioned how his mother loved him, above and beyond, providing material things for him. Throughout his narrative, Rashid talked about the role of his mother in his life. It seems like his relationship with his mother may have been relevant to his coping with stress and anger in adulthood. Additionally, the move to live with his grandfather after his mother passed away protected Rashid from a peer group who engaged in risky behaviors.

### Hakim: Role of Poverty in Shaping Men’s Abilities to Cope with Stress and Conflict

Hakim is 34-year old and married. He dropped out of school sometime in primary school. He works in the informal sector and sometimes has a challenge in finding work.

Hakim grew up with his maternal uncle and aunt, and a few of his half-siblings. All of his five siblings have different fathers. Both of his parents died when he was young. Prior to her death, his mother had arranged for Hakim to live with her brother. Hakim felt less valued than his uncle’s children. He had to rely on an older married sister for money to cover the costs of uniforms and school materials. He used to get less food than his cousins and sometimes went to sleep hungry. When he came back from school, his uncle made him sell mandazi (doughnuts) on the road before he could eat. When he misbehaved, his uncle locked him inside the house, beat him, and/or cut him. His uncle also regularly threatened to kick him out of the house and send him to live with his father’s family. Hakim described his uncle as an alcoholic, who frequently beat his wife. He mentioned hearing them fighting but could not intervene because he was too young.

Hakim was one of the few participants who, as an adult, did not condone the violence he experienced as a child.

Beating is not a teaching methodology. You can beat a child and yet he does not follow you and he can even become an extremist. Beating a child in that way is not good. You can just warn him/her verbally and he will develop good conducts and have a good life direction.

Hakim used to feel bad because he thought that if his mother was alive, he would have had a different life, one where he may have progressed further with school and would have not had to depend on informal jobs. Hakim left his uncle’s house in order to live with his father’s family when he was 10-year old.

Hakim currently experiences financial stress providing for his family. Like other participants, this financial stress seems to make him less able to cope with anger.

When I don’t get money, I must go home with stress because a child doesn’t know that his father has no money. So when he wakes up in the morning, he will demand money for breakfast bites or two to three hundred shillings school money because nowadays in school a child has to have two hundred shillings for porridge. So if you can’t even afford one-hundred shillings, a child will not understand you. So you are full of stress when you go home.

This financial stress made Hakim less likely to cope constructively with what he perceived as insults from others. He described that when he was stressed because he lacked money, he was more likely to perceive other’s actions as negative, and less likely to regulate his emotions without resorting to physical violence.

Something which can make me angry is when I have not left money at home so even when I walk on the road, I get angry so when I meet you on the way and you answer me badly, we can quarrel very easily… My anger is like that if you provoke me, I will just look at you and go aside. But there are things, which are not tolerable, and you may ask why this wants to see me as a fool? … If there is a club [blunt instrument] nearby you might hit him with it.

## Discussion

The focus of our analysis was on protective experiences or environments for violence in adulthood among participants who were exposed to adversity in childhood. The qualitative analysis had the following findings.

### Themes

#### Despite extensive adversity in childhood, early attachment to parents and parental acceptance could be conceptualized as protective for coping with stress and conflict with peers in adulthood.

Men in the study who described loving, accepting relationships in early childhood had fewer issues coping with stress and interacting among their peers than men with similar life histories who did not have these types of relationships. Research has shown that children with a secure child-parent bond, especially in early childhood, are better able to control their negative emotions in stressful situations and less likely to develop internalizing and externalizing behavior problems (Guttmann-Steinmetz & Crowell, 2006). Additionally, these children are better able to empathize and cooperate with others, enabling them to form strong relationships with peers in the future. For instance, Sroufe (2005) found that attachment history provided the foundation for variation in early peer relationships through variations in expectations, problem solving skills, and affect regulation capacities (Sroufe et al., 2009).

Secure attachment in early childhood has been found to buffer stress from childhood adversity, enabling better emotional regulation, and socio-emotional and cognitive functioning (Thompson, 2014). In the narrative of Rashid, his strong relationship with his mother and feeling of security about love among his family members in his early childhood could have mitigated the sequelae of stress in childhood, enabling him to regulate his emotions, particularly his anger, without resulting to violence.

Living in and frequently moving among alternative care situations with extended family members was common in the sample. Though some attachment theory researchers see informal kinship-based fostering as a problem for child development because the child has relationships with many caregivers and does not develop secure attachment with one caregiver (Cassidy, 2008; Kobak & Madsen, 2008), some academics caution against using attachment theory indiscriminately. They argue that it reflects “specific, Western child-rearing practices and ideologies and is not applicable in other cultural contexts where maternal thinking and child attachment patterns differ” (Leinaweaver, 2014, p. 4).

An alternate framework that has been used is the parental-acceptance-rejection theory, which seeks to take into account the “cultural variance in the expression and subjective experience” of parental acceptance and warmth (Major, 2007, p. 132) “Recognizing that the ways in which parents express love and affection are in large part culturally determined, the theory states that parents’ behaviors and intentions must be understood in cultural context” (Major, 2007, p. 132). Similarly, how children attribute meaning to a particular act may also vary across cultures. A systematic review and meta-analyses using 66 studies in 22 countries on five continents found that both maternal and paternal acceptance is associated with the psychological adjustment of children across cultures (Khaleque & Rohner, 2012). However, there is less consistent work on caregiver acceptance outside of parent-child dyads, including kinship-based fostering.

There are a number of factors that influence whether specific instances of kinship-based fostering would be harmful or beneficial. This is made even more complicated because, due to different life circumstances, children who have lived in kinship care typically live in multiple different families throughout their childhood and they live in kinship care for a number of reasons. For instance, we found that though Rashid faced economic hardship living in the village with his grandfather, it was more beneficial for him than living in a child-headed household with his sister. Even though his father reportedly had plenty of money, Rashid had a worse experience living with his father because he was not allowed to pursue an education and was not treated like part of the family. Leinaweaver (2014) argues that “more research is needed to understand the conditions under which informal kinship-based foster supports vulnerable children and families” (Leinaweaver, 2014 p. 5).

#### Certain key life turning points can provide a protective or risky context against violent behavior in adulthood.

In the narratives, there were a number of turning points that had an effect on the behavior of participants. The men were often moved to the homes of different kin due to circumstances such as the death of a parent, opportunity for a better school, or behavioral problems. These physical moves provided changes in their social and physical environment, which oftentimes had an impact on their trajectory of behaviors. For instance, Rashid experienced a turning point away from behaviors such as stealing and substance use. Though his mother died and he lost her support, he saw leaving the context of the informal settlement as a new start, away from his peers who went on to be killed engaging in risky activities.

Turning points are often studied in the context of life course criminology. According to Sampson and Laub (2005), there are a number of mechanisms that underlie the developmental process of leading to desistance (a sustained absence of perpetration of crime). One of the main mechanisms is a new situation that separates past from present. Additionally, there are more positive outcomes when the situations also provide supervision, monitoring, and a new structure of routine activities, new possibilities for social support and growth, and chance for identity transformation (Sampson & Laub, 2005). For Rashid, his mother’s death and his subsequent move to live with his grandfather provided a definitive change from the past, as well as new structure to his routine activities. Though he was severely beaten by one teacher and did not receive much supervision and monitoring from his elderly grandfather, he received social support from the teachers who understood his circumstances and encouraged him to continue with his education.

Sampson and Laub (2005) also argue that human agency is an important element in constructing trajectories over the life course. They quote Abbot (1997) as saying “A major turning point has the potential to open a system the way a key has the potential to open a lock action is necessary to complete the turning.” In the case of Rashid, with all that was stacked against him, including his poverty, an ailing grandfather and severe physical abuse from teachers, he chose to put his effort into continuing his education.

#### Economic stress decreased men's ability to cope with anger and conflict among peers

Almost all of the men faced some economic stress during adulthood. They felt pressure to establish a homestead of their own and provide for their families. This is not surprising, given that the sample is from a district where many men are from a low socioeconomic status. However, in many cases, especially in cases where they had a predisposition to violence, this pressure seemed to exacerbate the effect of childhood adversity on their ability to cope with stress and anger in adulthood. A number of studies have found that lack of economic opportunities, unemployment, and economic stress are associated with both IPV and youth PV (Wilkins et al., 2014).

Studies have found that childhood adversity is not only associated with adult poverty, but also with a reduced capacity to effectively manage behavior, emotions, and interpersonal relationships without reverting to violence (Bunting et al., 2018; Ford, 2005). The Catalyst Model of Aggression hypothesizes that individuals develop a predisposition to violence as a result of personal characteristics (e.g., genes, personality) and historical environmental factors (e.g., exposure to ACEs in childhood). Individuals with a high level of predisposition to violence are more likely to respond to environmental triggers (e.g., social interactions, stressors) with violence (Ferguson, 2010). In this model, economic stress can increase the likelihood of violence in individuals who are already prone to violent behavior. Though economic stress was widely experienced in the study, it was a catalyst for Hakkim, since he already had a high level of predisposition to violence based on his personal characteristics and experiences in childhood.

This fits with neurobiological evidence, where adverse experiences in childhood have been found to decrease one’s ability to recover from negative affect without excessive inhibition and a lack of restraint of impulsive/aggressive behavior (Ford, 2005). Therefore, it is possible that among the participants exposed to child adversity, economic stress leads to excessive lack of restraint manifested in disregard of social conventions, impulsivity, and poor risk assessment. This may then lead to perceiving a peer’s behavior as threatening and subsequently behaving aggressively, such as Hakim hitting community members in the street with whatever blunt instrument he could find because he perceived that the man provoked him.

### Limitations

Some limitations of this study should be noted, while we purposively sampled from a list of participants who had the highest probability of perpetrating interpersonal violence, based on the quantitative person-centered analysis, oftentimes participant experiences as related in the in-depth interviews did not correspond to how those experiences were reported on the behavioral survey. This could be due to differences in how questions of perpetration were asked in the two methods, differences in how comfortable participants felt reporting using the two methods, or differences in recall. This could also be due to interviewers getting a chance to build rapport with participants throughout the in-depth interviews. Since the data were collected in Kiswahili and then translated into English for analysis, some information and meaning may have been lost in the translation process. To guard against this, we compared the field notes to from the research assistants to the translated transcripts. Also, knowledge and context could have been lost in the translation process. However, we took measures to ensure that this did not happen, including frequent consulting with study research assistants and translator. The study also has the limitation of having employed a single coder. However, the codebook was reviewed prior to coding with the Tanzania site PI to ensure that the codes were clear and relevant to the interview text. Finally, the study was focused on the experiences of men, thus women’s experience with early childhood exposure to adversity and later perpetration of violence are not captured in these findings.

The guide for the in-depth interviews was semi-structured, and we used an inductive approach focused on experiences of violence, stressors in adulthood, and interpersonal violence perpetration as an adult. This approach did not reflect the traditional narrative approach that emphasizes the active construction of life stories through the interplay between interviewer and interviewee (Miller, 2000). This was because the narrative approach was selected after the data had been collected. Relatedly, since the study was more exploratory and the major themes were not identified until the analysis, we were not able to probe on and analyze how the main themes interacted across the life course.

### Future Research and Programming

More exploration is needed on how the study themes interact and possibly moderate the relationship between exposure to adversity in childhood and coping with stress and anger in adulthood. Relatedly, exploration of the experience of those who were exposed to adversity in childhood is needed to further elucidate the individual and social mechanisms that mediate the relationship between exposure to childhood adversity and perpetration of interpersonal violence in adulthood. In particular, there needs to be additional research on mechanisms for promoting attachment and caregiver warmth among children in informal kinship-based fostering.

Further research is also needed on turning points for intervention to prevent risky behaviors among adolescents and youths who were previously exposed to adversity in childhood, particularly what makes children get involved in gang-like groups and at what point are children involved in risky behaviors more or less likely to transition away from this type of behavior.

For programming, findings reflect that early attachment to caregivers among children who are the most vulnerable to childhood adversity could be protective against risky behaviors in adulthood. Therefore, additional research could inform whether evidenced-based interventions such as attachment and biobehavioral catch-up (ABC) can be effective in this population. ABC interventions are targeted to caregivers in vulnerable families whose children have experienced neglect, physical abuse, domestic violence, and placement instability. The goals of ABC is to (a) increase caregiver nurturance, sensitivity, and delight, (b) decrease caregiver frightening behaviors, (c) increase child attachment security and decrease disorganized attachment, and (d) increase child behavioral and biological regulation (Dozier & Bernard, 2017). A recent systematic review of 10 RCTs found that ABC is effective when implemented with child-welfare-involved children, in improving affect regulation, improving externalizing and internalizing behaviors, increasing normative developmental functioning, and attachment quality (Grube & Liming, 2018). However, none of these studies were conducted in low- and middle-income country contexts.

## Conclusion

Our findings contextualize, among high-risk men in Dar es Salaam who have been exposed to adversity in childhood, how different factors may affect the behavior of perpetration of interpersonal violence. These findings provide important contextual information on the risk and protective factors for interpersonal violence spanning from childhood to adulthood. Particularly, we found that parental acceptance and attachment at a young age is key in protecting against violent behavior in adulthood. This highlights the importance of parenting and child development interventions in this context, both for the primary prevention of child adversity and for promoting resilience and mitigating the effects of childhood adversity that put men at risk for perpetration of interpersonal violence in adulthood.

## Author Biographies

**Susannah Zietz**, PhD, MPH, is a postdoctoral scholar with the Center for Child and Family Policy. She graduated May 2019 with a PhD. in health behavior in the Gillings School of Global Public Health at UNC Chapel Hill. She is currently working on the Parenting Across Cultures study and is interested in the relationship between exposure to adversity in childhood and aggression and health risk behaviors in adolescence and adulthood. She is specifically interested in indicators of self-regulation as possible mediators and positive parenting and parental warmth a possible moderators of these relationships.

**Lusajo Kajula**, MPhil, PhD, is a research analyst with the UNICEF Office of Research-Innocenti. Her research interests lie in adolescent and youth sexual health, Intimate Partner Violence (IPV), Violence against Children (VAW), HIV related stigma, as well as parenting and family interactions. Since 2002, Lusajo has been involved in several international collaborations that addressed adolescent and youth sexual behaviour in Sub-Saharan Africa. Her past research projects included being site PI for an intervention trial that looked at a population of young men in 60 camps, parents’ and teacher’s communication with youth about HIV-related sexual behaviours in Tanzania as well as social and cultural factors associated with concurrent sexual partnerships in Tanzania amongst others. At Innocenti, she coordinates a study on Cash Plus for Adolescents in Tanzania and supports qualitative research more generally in the Transfer Project.

**Sandra Martin**, PhD, is a professor and associate chair for research in the Department of Maternal and Child Health in the Gillings School of Global Public Health at the University of North Carolina at Chapel Hill. She is currently working with colleagues on studies of: sexual misconduct on college campuses; sexual assault prevention programs for the military; school -based programs to prevent sex trafficking of children; how gender identity and sexual orientation are associated with intimate partner violence victimization and perpetration; and how housing tax policies are related to the risk of intimate partner violence and child maltreatment.

**Beth Moracco**, PhD, MPH, is an associate professor in the Department of Health Behavior at UNC Chapel Hill. She is also the associate director of the UNC Injury Prevention Research Center. Her current research includes evaluations of a community-based sexual violence prevention program, electronic filing of domestic violence protective orders (DVPOs), and firearm confiscation and surrender policies in civil domestic violence cases. She has published more than 90 peer-reviewed articles, book chapters, and invited commentaries.

**Meghan Shanahan**, PhD, MPH, is an assistant professor in the Department of Maternal and Child Health and a research scientist at the UNC Injury Prevention Research Center. Her research focuses on adverse events that potentially influence these trajectories and prevent children from realizing their full potential. Child maltreatment is one obvious insult to the health and development of children and, therefore, documenting the magnitude, etiology, and impact of child maltreatment has been a focus of Dr. Shanahan’s work. Examining prevention strategies to reduce child abuse and neglect has become another main focal point of her research. She has evaluated prevention strategies at both the family and the policy level in North Carolina. Dr. Shanahan is also interested in examining the impact of prescription and illicit opioid use on parenting and, subsequently, child development, as well as the effects of experiencing intimate partner violence. She is also committed to translating research into tangible products and policies that have an impact on children and families.

**Suzanne Maman**, PhD, MPH, is a professor of Health Behavior and associate dean for global health at the Gillings School of Global Public Health at the University of North Carolina Chapel Hill. Trained as a social scientist in public health, she has 20 years of research experience related to HIV/AIDS and gender-based violence in sub-Saharan Africa. Dr. Maman’s work focuses on two aspects of the association between HIV and violence: (a) violence as a barrier to implementing HIV risk reduction strategies and (b) violence as an outcome of HIV testing and HIV status disclosure to sexual partners. As associate dean for global health, Maman works closely with colleagues within the Gillings School’s Research, Innovation and Global Solutions unit to integrate global initiatives into innovation, entrepreneurship and research, and lead programming that supports the School’s domestic and international students.
